# Different effects of high-fat and high-sucrose diets on the physiology of perivascular adipose tissues of the thoracic and abdominal aorta

**DOI:** 10.1080/21623945.2021.1965333

**Published:** 2021-09-13

**Authors:** Tsukasa Sasoh, Hirona Kugo, Yuya Kondo, Kento Miyamoto, Momoka Minami, Mayo Higashihara, Hirokazu Kawamoto, Fumiaki Takeshita, Tatsuya Moriyama, Nobuhiro Zaima

**Affiliations:** a Department of Applied Biological Chemistry, Graduate School of Agriculture, Kindai University, Nara, Japan; b OHKI PHARMACEUTICAL CO., LTD., Tokyo, Japan; c Agricultural Technology and Innovation Research Institute, Kindai University, Nara, Japan

**Keywords:** Perivascular adipose tissue, vascular diseases, high-fat diet, high-sucrose diet, aneurysm

## Abstract

Vascular diseases such as atherosclerosis and aneurysms are associated with diet. Perivascular adipose tissue (PVAT) was reportedly involved in the regulation of vascular functions. It is suggested that imbalanced diets can cause PVAT inflammation and dysfunction as well as impaired vascular function. However, the association between diets and PVAT are not clearly understood. Here, we showed that a high-fat and a high-sucrose diet affected PVAT at different sites. A high-fat diet induced increased number of large-sized lipid droplets and increased CD (Cluster of differentiation) 68+ macrophage- and monocyte chemotactic protein (MCP)-1-positive areas in the abdominal aortic PVAT (aPVAT). In addition, a high-fat diet caused decreased collagen fibre-positive area and increased CD68+ macrophage- and MCP-1-positive areas in the abdominal aorta. In contrast, a high-sucrose diet induced increased number of large-sized lipid droplets, increased CD68+ macrophage- and MCP-1-positive areas, and decreased UCP-1 positive area in the thoracic aortic PVAT (tPVAT). A high-sucrose diet caused decreased collagen fibre-positive area and increased CD68+ macrophage- and MCP-1-positive areas in the thoracic aorta. These results could be attributed to the different adipocyte populations in the tPVAT and aPVAT. Our results provide pathological evidence to improve our understanding of the relationship between diet and vascular diseases.

## Introduction

Vascular diseases such as atherosclerosis and aneurysms have been reported to be associated with western diets [1]. Recent studies have also suggested a possible relationship between vascular diseases and perivascular adipose tissue (PVAT), the adipose tissue surrounding the aorta, with the exception of the cerebral vasculature. The major physiological role of PVAT was previously considered to be providing mechanical support to blood vessels; however, recent studies have recognized it as a physiologically and metabolically active endocrine tissue that has important effects on vascular function and diseases [2,3]. Similar to other adipose tissues, PVAT secretes both pro-inflammatory and anti-inflammatory adipocytokines [4]. The proximity of PVAT with the aorta has led to speculations that vasoactive adipocytokines produced from PVAT could affect the vascular function in a paracrine manner [5]. Adipocytokine production in PVAT is associated with adipocyte size [6,7].

Under normal conditions, PVAT exerts an anti-inflammatory effect and shows a relatively low secretion of pro-inflammatory cytokines [7]. However, excessive accumulation of triglycerides in PVAT causes the enlargement of adipocytes due to excess lipid droplets, resulting in inflammation of PVAT, accompanied by increased macrophage infiltration [8–11]. Inflammation in PVAT reduces its secretion of anti-inflammatory adiponectin [12], while increasing its secretion of pro-inflammatory cytokines such as interleukin (IL)-6, IL-8, and especially monocyte chemotactic protein (MCP)-1 [8–11]. In vascular diseases such as aortic aneurysm, atherosclerosis, and vasculitis syndrome, PVAT inflammation has been shown to promote pro-inflammatory cytokine production, leading to infiltration of inflammatory cells (macrophages, neutrophils, and T cells) into blood vessels from the adventitial side [13–17]. The consequent increase in the number of inflammatory cells in the vessel walls promote matrix fragmentation of the aortic wall by increasing matrix metalloproteinase (MMP) expression and activity [18–20]. These inflammatory processes can be attributed to functional changes in PVAT [2,3,21,22].

Since dietary fats and sucrose are converted to triglycerides, fat- or sucrose-rich diets can cause hypertrophy of adipocytes, resulting in adipose tissue inflammation [23]. Excessive intake of fat and sucrose has been shown to have different effects on the pathology of vascular and metabolic diseases [24–27]. However, the effects of high-fat (HF) and high-sucrose (HS) diets on PVAT remain unclear. Investigating the effects of diet on PVAT function could help improve our understanding of the relationship between diet and vascular diseases. In this study, we investigated the effects of high-fat and high-sucrose diets on the aortic wall and PVAT of the thoracic aorta and abdominal aorta.

## Results

### Effects of high-fat and high-sucrose diets on body weight, food intake, and serum glucose, triglyceride, and total cholesterol levels

**Figure 1.**
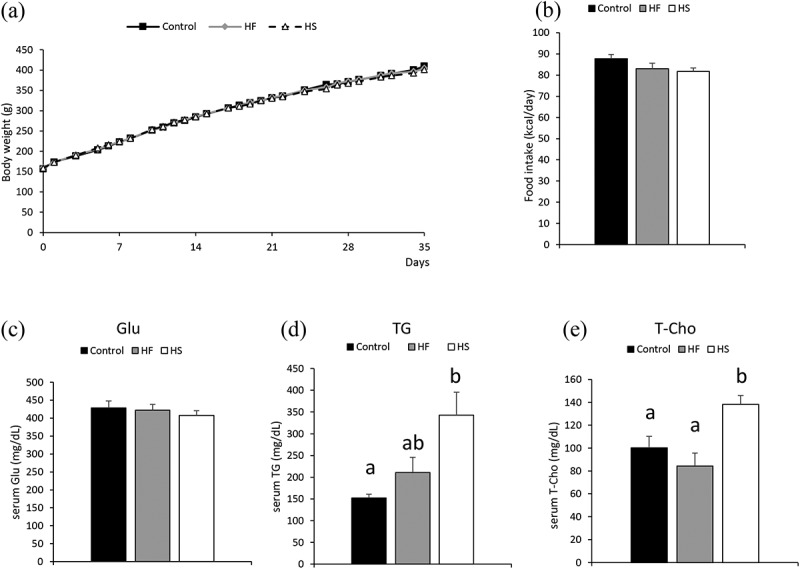
Effects of high-fat and high-sucrose diets on body weight, food intake, serum glucose, serum triglyceride and serum total cholesterol levels. (a) Body weight, (b) food intake, (c) serum glucose, (d) serum TG and (e) serum T-Cho in the three experimental groups. Data are presented as the mean ± standard error of mean (S.E.M). Values with different letters are significantly different (p < 0.05)

Body weight and food intake did not differ between the three groups (Figure 1(a,b)). Additionally, serum glucose levels were not different between the three groups (Figure 1(c)). In contrast, serum triglyceride (TG) levels were significantly higher in the HS group than in the control group (Figure 1(d)). Furthermore, serum total cholesterol (T-Cho) was significantly increased in the HS group, compared to the control and HF groups (Figure 1(e)). Serum leptin level was significantly increased in the HF group compared to the control and HS groups (Supplementary Figure 1 (a)). Serum insulin level was significantly increased in the HS group compared to the control and HF groups (Supplementary Figure 1 (b)).

### Effects of high-fat and high-sucrose diets on lipid droplets in PVAT

**Figure 2.**
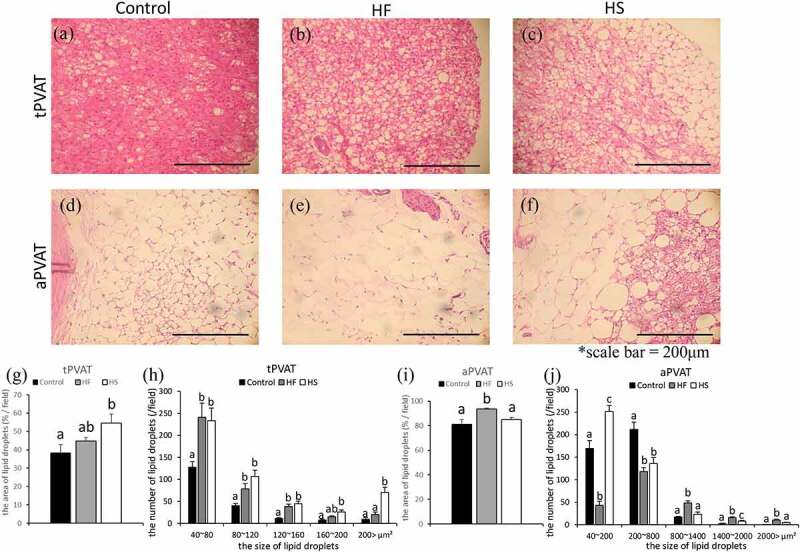
Effects of high-fat and high-sucrose diets on lipid droplets in PVAT. (a-c) Represented haematoxylin-eosin (H&E) staining of the tPVAT and (d-f) represented (H&E) staining of the aPVAT in the three experimental groups. (g) Lipid droplets ratio of the tPVAT, (h) lipid droplets number per 1 field (200,000μm^2^) of the tPVAT, (i) lipid droplets rate of the aPVAT and (j) lipid droplets number per 1 field (200,000μm^2^) of the aPVAT in the three experimental groups. Scale bar = 200 μm. Data are presented as the mean ± standard error of mean (S.E.M). Values with different letters are significantly different (p < 0.05)

Levels of lipid accumulation were investigated in the thoracic aortic PVAT (tPVAT) and abdominal aortic PVAT (aPVAT) (Figure 2). The area of lipid droplets in the tPVAT was significantly increased in the HS group, compared to the control group (Figure 2(a-c,g)). In the aPVAT, the area of lipid droplets was significantly increased in the HF group, compared to the control and HS groups (Figure 2(d-f,i)).

We then measured the size of each lipid droplet and compared the number of lipid droplets between the groups. In tPVAT, the number of lipid droplets with areas ranging from 40–80 μm^2^, 80–120 μm^2^, and 120–160 μm^2^ was significantly higher in the HF and HS groups than in the control group (Figure 2(a-c,h)). The number of lipid droplets with an area ranging from 160–200 μm^2^ was significantly higher in the HS group than in the control group (Figure 2(a-c,h)). Finally, the number of lipid droplets with an area larger than 200 μm^2^ was significantly higher in the HS group than in the control and HF groups (Figure 2(a-c,h)). In aPVAT, the number of lipid droplets with an area ranging from 40–200 μm^2^ was significantly lower in the HF group than in the control and HS groups, while the number of such lipid droplets was significantly higher in the HS group than in the control and HF groups (Figure 2(d-f,j)). The number of lipid droplets with an area ranging from 200–800 μm^2^ was significantly lower in the HF and HS groups than in the control group (Figure 2(d-f,j)). The number of lipid droplets with an area ranging from to 800–1400 μm^2^ was significantly higher in the HF group than in the control and HS groups (Figure 2(d-f,j)). The number of lipid droplets with an area ranging from 1400–2000 μm^2^ was significantly higher in the HF groups than in the control and HS groups; the number of such lipid droplets was significantly higher in the HS group than in the control group (Figure 2(d-f,j)). Finally, the number of lipid droplets with an area larger than 2000 μm^2^ was significantly higher in the HF group than in the control and HS groups (Figure 2(d-f,j)).

### Effects of high-fat and high-sucrose diets on UCP-1 in PVAT

**Figure 3.**
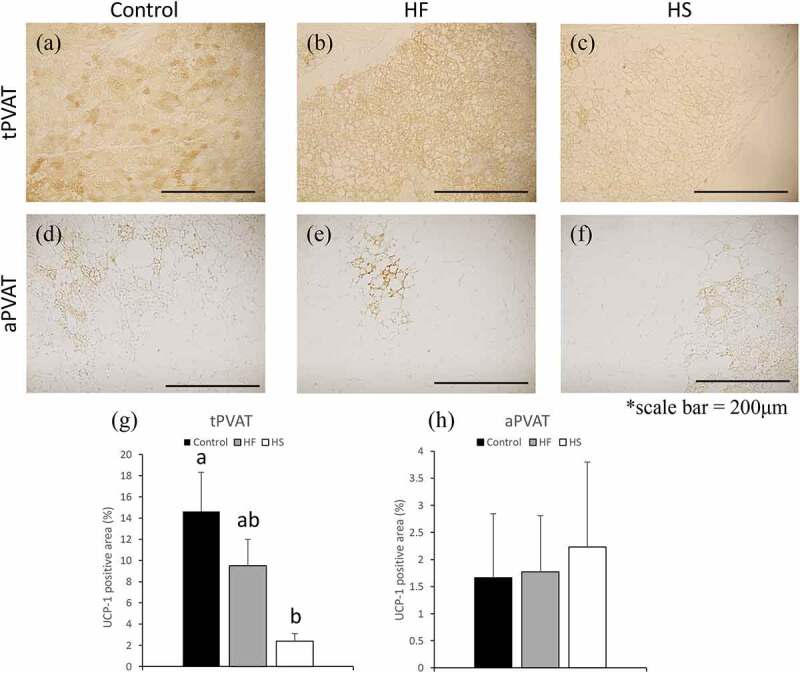
Effects of high-fat and high-sucrose diets on UCP-1 in PVAT. (a-c) Represented UCP-1 immunostaining of the tPVAT and (d-f) represented UCP-1 immunostaining of the aPVAT in the three experimental groups. (g) Quantification of UCP-1 positive areas of the tPVAT and (h) quantification of UCP-1 positive areas of the aPVAT, in the three experimental groups. Scale bar = 200 μm. Data are presented as the mean ± standard error of mean (S.E.M). Values with different letters are significantly different (p < 0.05)

Areas that stained positive for uncoupling protein (UCP)-1 in tPVAT and aPVAT were compared among the three groups. In tPVAT, UCP-1 was ubiquitously stained in all groups (Figure 3(a-c)). However, UCP-1-positive areas were significantly decreased in the HS group, compared to the control group (Figure 3(a-c,g)). In aPVAT, UCP-1 was partially stained in all groups (Figure 3(d-f)), with no significant difference among the three groups (Figure 3(d-f,h)).

### Effects of high-fat and high-sucrose diets on inflammatory markers in PVAT

**Figure 4.**
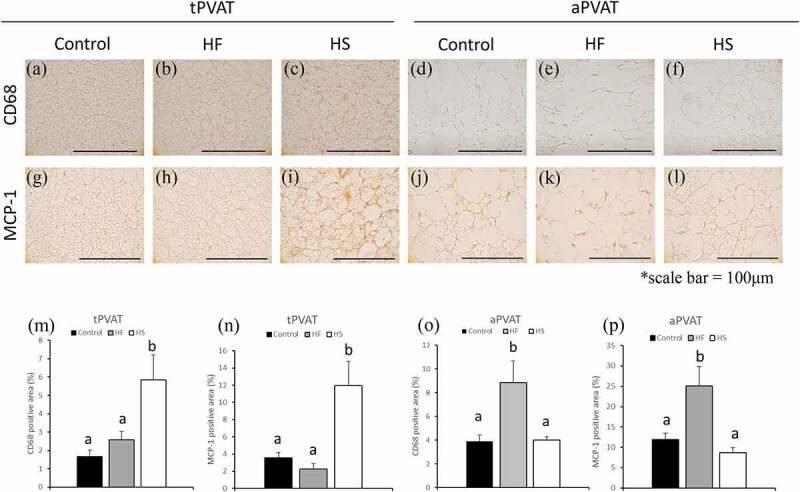
Effects of high-fat and high-sucrose diets on macrophage and MCP-1 in PVAT. (a-c) Represented CD68 immunostaining of the tPVAT, (d-f) represented CD68 immunostaining of the aPVAT, (g-i) represented MCP-1 immunostaining of the tPVAT and (j-l) represented MCP-1 immunostaining of the aPVAT in the three experimental groups. (m) Quantification of CD68 positive areas of the tPVAT, (n) quantification of MCP-1 positive areas of the tPVAT, (o) Quantification of CD68 positive areas of the aPVAT and (p) quantification of MCP-1 positive areas of the aPVAT in the three experimental groups. Scale bar = 100 μm. Data are presented as the mean ± standard error of mean (S.E.M). Values with different letters are significantly different (p < 0.05)

Areas that stained positive for CD (Cluster of differentiation) 68+ macrophages and MCP-1 in tPVAT and aPVAT were compared among the three groups. In tPVAT, CD68-positive areas were significantly higher in the HS group than in the control and HF groups (Figure 4(a-c,m)). In aPVAT, CD68-positive areas were significantly higher in the HF group than in the control and HS groups (Figure 4(d-f,o)). In tPVAT, MCP-1-positive areas were significantly higher in the HS group than in the control and HF groups (Figure 4(g-i,n)). In aPVAT, MCP-1-positive areas in the HF group were significantly higher than those in the control and HS groups (Figure 4(j-l,p)).

**Figure 5.**
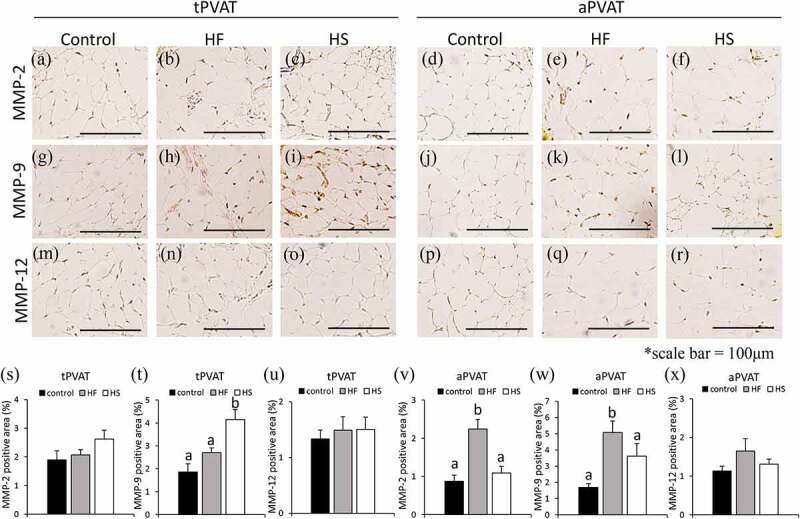
Effects of high-fat and high-sucrose diets on MMP-2, MMP-9 and MMP-12 in PVAT. (a-c) Represented MMP-2 immunostaining of the tPVAT, (d-f) represented MMP-2 immunostaining of the aPVAT, (g-i) represented MMP-9 immunostaining of the tPVAT and (j-l) represented MMP-9 immunostaining of the aPVAT, (m-o) represented MMP-12 immunostaining of the tPVAT and (p-r) represented MMP-12 immunostaining of the aPVAT in the three experimental groups. (s) Quantification of MMP-2 positive areas of the tPVAT, (t) quantification of MMP-9 positive areas of the tPVAT, (u) quantification of MMP-12 positive areas of the tPVAT, (v) quantification of MMP-2 positive areas of the aPVAT, (w) quantification of MMP-9 positive areas of the aPVAT and (x) quantification of MMP-12 positive areas of the aPVAT in the three experimental groups. Scale bar = 100 μm. Data are presented as the mean ± standard error of mean (S.E.M). Values with different letters are significantly different (p < 0.05)

Areas that stained positive for MMPs in tPVAT and aPVAT were compared among the three groups. In tPVAT, positive areas for MMP-2 and MMP-12 were not significantly different between three groups (Figure 5(a-c,m-o,s,u)). In tPVAT, positive areas for MMP-9 were significantly higher in the HS group than in the control and HF groups (Figure 5(g-i,t)). In aPVAT, positive areas for MMP-2 and MMP-9 were significantly higher in the HF group than in the control and HS groups (Figure 5(d-f,j-l,v,w)). In aPVAT positive areas for MMP-12 were not significantly different between the three groups (Figure 5(p-r,x)).

Areas that stained positive for resistin, TNF-α, adiponectin and angiotensinogen in tPVAT and aPVAT were compared among the three groups. In tPVAT, positive areas for resistin were significantly higher in the HS group than in the control group (Supplementary Figure 2 (a-c, m)). In tPVAT, positive areas for TNF-α and adiponectin were not significantly different between the three groups (Supplementary Figure 2 (g-i, n) and Supplementary Figure 3 (a-c, m)). In tPVAT, positive areas for angiotensinogen were significantly higher in the HF group than in the control group (Supplementary Figure 3 (g-i, n)). In aPVAT, positive areas for resistin and TNF-α were significantly higher in the HF group than in the control and HS groups (Supplementary Figure 2 (d-f, j-l, o, p)). In aPVAT, positive areas for adiponectin were significantly lower in the HS group than in the control and HF groups (Supplementary Figure 3 (d-f, o)). In aPVAT, positive areas for angiotensinogen were significantly higher in the HF group than in the control group (Supplementary Figure 3 (j-l, p)).

### Effects of high-fat and high-sucrose diets on the thoracic and abdominal aortae

The collagen fibre-positive areas of the thoracic and abdominal aortae were compared among the three groups. In the thoracic aorta, the collagen fibre-positive areas were significantly smaller in the HS group than in the control and HF groups (Figure 5(a-c,s)). In contrast, in the abdominal aorta, the collagen fibre-positive areas were significantly smaller in the HF group than in the control and HS groups (Figure 5(d-f,v)).

**Figure 6.**
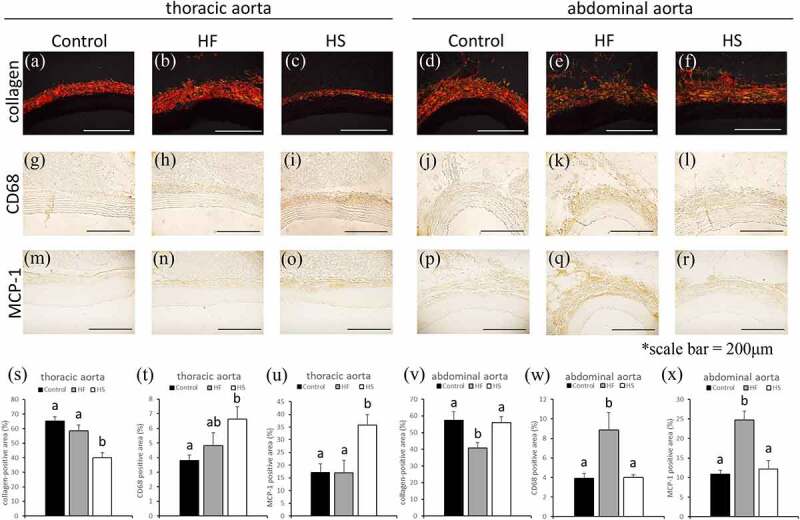
Effects of high-fat and high-sucrose diets on the thoracic and abdominal aorta. (a-c) represented picrosirius red (PSR) staining of the thoracic aorta, (d-f) represented (PSR) staining of the abdominal aorta, (g-h) represented CD68 immunostaining of the thoracic aorta, (j-l) Represented CD68 immunostaining of the abdominal aorta, (m-o) represented MCP-1 immunostaining of the thoracic aorta and (p-r) represented MCP-1 immunostaining of the abdominal aorta in the three experimental groups. (s) Collagen positive areas quantification of the thoracic aorta, (t)quantification of CD68 positive areas of the thoracic aorta, (u) quantification of MCP-1 positive areas of the thoracic aorta, (v) collagen positive areas quantification of the abdominal aorta, (w) quantification of CD68 positive areas of abdominal aorta and (x) quantification of MCP-1 positive areas of abdominal aorta in the three experimental groups. Scale bar = 200 μm. Data are presented as the mean ± standard error of mean (S.E.M). Values with different letters are significantly different (p < 0.05)

Additionally, we compared the CD68+-positive macrophages and MCP-1-positive areas in the thoracic and abdominal aorta among the three groups. In the thoracic aorta, CD68-positive areas were significantly larger in the HS group than in the control group (Figure 6(g-i,t)). In the abdominal aorta, CD68-positive areas were significantly larger in the HF group than in the control and HS groups (Figure 6(j-l,w)). Similarly, in the thoracic aorta, MCP-1-positive areas were significantly larger in the HS group than in the control and HF groups (Figure 6(m-o,u)). In the abdominal aorta, MCP-1-positive areas were significantly larger in the HF group than in the control and HS groups (Figure 6(p-r,x)).

**Figure 7.**
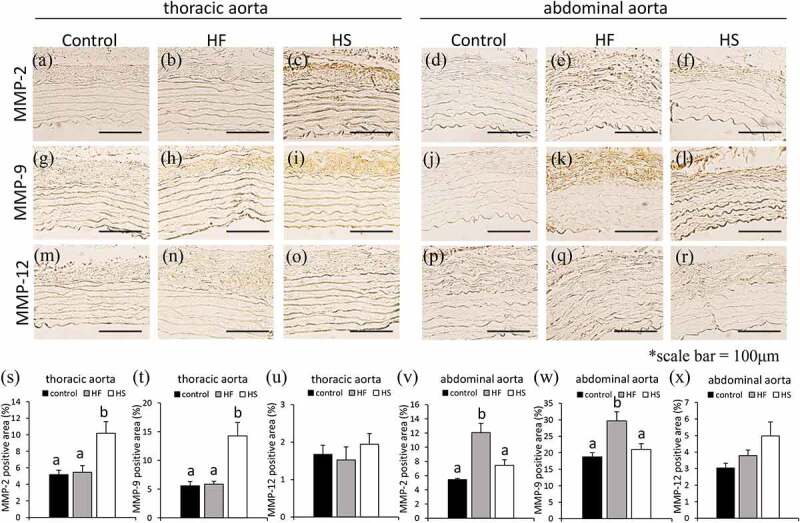
Effects of high-fat and high-sucrose diets on MMPs in the thoracic and abdominal aorta. (a-c) represented MMP-2 immunostaining of the thoracic aorta, (d-f) represented MMP-2 immunostaining of the abdominal aorta, (g-h) represented MMP-9 immunostaining of the thoracic aorta, (j-l) Represented MMP-9 immunostaining of the abdominal aorta, (m-o) represented MMP-12 immunostaining of the thoracic aorta and (p-r) represented MMP-12 immunostaining of the abdominal aorta in the three experimental groups. (s) Quantification of MMP-2 positive areas of the thoracic aorta, (t) quantification of MMP-9 positive areas of the thoracic aorta, (u) quantification of MMP-12 positive areas of the thoracic aorta, (v) quantification of MMP-2 positive areas of the abdominal aorta, (w) quantification of MMP-9 positive areas of abdominal aorta and (x) quantification of MMP-12 positive areas of abdominal aorta in the three experimental groups. Scale bar = 100 μm. Data are presented as the mean ± standard error of mean (S.E.M). Values with different letters are significantly different (p < 0.05)

We compared the MMPs in the thoracic and abdominal aortae among the three groups. In the thoracic aorta, positive areas for MMP-2 and MMP-9 were significantly higher in the HS group than in the control and HF groups (Figure 7(a-c,g-i,s,t)). In the thoracic aorta, positive areas for MMP-12 were not significantly different between the three groups (Figure 7(m-o,u)). In the abdominal aorta, positive areas for MMP-2 and MMP-9 were significantly higher in the HF groups than in the control and HS groups (Figure 7(d-f,j-l,v,w)). In the abdominal aorta, positive areas for MMP-12 were not significantly different between the three groups (Figure 7(p-r,x)).

We compared the resistin, TNF-α, adiponectin and angiotensinogen positive areas in the thoracic and abdominal aortae among the three groups. In the thoracic aorta, positive areas for resistin, TNF-α and angiotensinogen were not significantly different between three groups (Supplementary Figure 4 (a-c, g-i, m, n) and Supplementary Figure 5 (g-i, n)). In the thoracic aorta, positive areas for adiponectin were significantly lower in the HF and HS groups than in the control group (Supplementary Figure 5 (a-c, m)). In the abdominal aorta, positive areas for resistin and adiponectin were not significantly different between the three groups (Supplementary Figure 4 (d-f, o) and Supplementary Figure 5 (d-f, o)). In the abdominal aorta, positive areas for TNF-α were significantly higher in the HF group than in the control group (Supplementary Figure 4 (j-l, p)). In abdominal aorta, positive areas for angiotensinogen were significantly higher in the HF and HS groups than in the control group (Supplementary Figure 5 (j-l, p)).

## Discussion

Unbalanced diets can cause accumulation of excessive triglycerides in adipose tissue, leading to inflammation and dysfunction of vascular functions [1–3]. In this study, we found characteristic differences in the effects of high-fat and high-sucrose diets on PVAT and aortae.

The composition of adipocyte species in PVAT is known to differ depending on the site [28]. The major components of PVAT in the surroundings of the coronary artery and thoracic aorta are brown adipocytes [29,30]. In contrast, PVAT surrounding the mesenteric artery and abdominal aorta mainly comprises white adipocytes [30]. Here, we found that UCP-1, a marker of brown adipocytes, was ubiquitously stained in tPVAT but was only partially stained in aPVAT. These findings are consistent with previous reports [30].

In tPVAT, increased levels of lipid droplets were observed in both the HF and HS diet groups. However, the number of larger lipid droplets (with area larger than 200 μm^2^) were significantly increased only in the HS group. In addition, decreased levels of UCP-1 were observed in the HS group, suggesting that HS diets may decrease the thermogenic function of brown adipocytes. Moreover, increased levels of macrophages and MCP-1 were observed in the tPVAT of the HS group. Brown adipocytes are less likely to cause inflammation and increase in macrophage infiltration than white adipocytes, due to less lipid accumulation [14,30]. However, it has also been reported that ‘whitening’ brown adipocytes can cause a decrease in heat production and an increase in inflammation [31–33]. In tPVAT, HS diet induced increased level of MMP-9 which degrade extracellular matrix such as elastin and collagen fibres. In tPVAT, macrophages and hypertrophic adipocytes may induce MMPs secretion and promote PVAT dysfunction in the HS group. In tPVAT, resistin levels were also increased in HS fed rats. Dysfunctional PVAT reportedly induces vascular dysfunction by increased secretion of pro-inflammatory adipokines such as TNF-α and resistin [34]. Our results suggest that the HS diet may causes ‘whitening’ of tPVAT, leading to dysfunction and inflammation in the tPVAT and aortic wall. Brown adipocytes activate heat-producing functions and improve hypertriglyceridaemia and hypercholesterolemia [35–37]. The qualitative changes of brown adipocytes in tPVAT in the HS group might therefore be associated with hyperlipidaemia and hypercholesterolemia.

In contrast, aPVAT was sensitive to the HF diet but not to the HS diet. The HF diet induced an increase in the number of larger lipid droplets in aPVAT. Hypertrophy of lipid droplets in white adipocytes has been reported to increase the production of macrophages and inflammatory adipocytokines [8]. Here, we observed increased macrophage infiltration, MCP-1 and MMPs levels in aPVAT in the HF group, suggesting that HF diets could cause adipocyte hypertrophy in aPVAT. In addition, our results suggest that aPVAT dysfunction can be associated with the increased levels of resistin, TNF-α, and angiotensinogen. Angiotensinogen is involved in hypertension as a precursor of angiotensin II [38,39]. HF diets might increase blood pressure and cause vascular injury by increasing these factors in aPVAT. In the HF group, we found an increase in the area that stained positive for malondialdehyde (MDA), an oxidative stress maker, indicating that HF diets could cause inflammatory changes in the aPVAT (Supplementary Figure 6).

Qualitative changes in PVAT, such as hypertrophy of adipocytes and macrophage infiltration, can cause macrophage infiltration in the adjacent adventitial region of the aorta as well, through the secretion of inflammatory cytokines such as IL-6, IL-8 and MCP-1 [13–17]. We observed increased levels of macrophages, MCP-1 and MMPs in the tPVAT of the HS diet group and in the aPVAT of the HF diet group. In addition, decreased levels of collagen fibres were observed in the thoracic aorta of the HS diet group and in the abdominal aorta of the HF diet group. Collagen fibres play a central role in the maintenance of the aortic structure [40]. Degeneration of collagen fibres is associated with vascular diseases, especially aneurysms [41]. HF diets have been reported to increase the risk of abdominal aortic aneurysm (AAA) development [14,25,42,43]. In contrast, the HS diet does not affect the risk of development and rupture of AAA [26]. Previous studies have suggested a relationship between the dysfunction of PVAT and AAA [44–47]. HF diet-induced inflammatory changes in aPVAT might affect the development and rupture of AAA. It would be of great interest to investigate the relationship between HS diets and thoracic aortic aneurysms. However, there is a lack of scientific data in this field due to the absence of animal models for thoracic aortic aneurysms.

Effects of HF and HS diets on PVAT and the aortic wall are summarized in Table 1. Variation of inflammatory makers were mainly observed in the tPVAT and thoracic aorta of the HS group and in the aPVAT and abdominal aorta of the HF group. However, there were some factors that did not follow the trend to this variation. For example, angiotensinogen levels were increased in both tPVAT and aPVAT of the HF group. Further studies are needed to elucidate the mechanisms underlying the difference between HF and HS diets.

**Table 1.** Effects of high-fat and high-sucrose diets

(a) Effects of high-fat and high-sucrose diets on PVAT											
		Collagen	CD68	MCP-1	MMP-9	MMP-2	TNF-α	Resistin	Angiotensinogen	Adiponectin	MMP-12
tPVAT	HF	N.D.	→	→	→	→	→	→	↑	→	→
	HS	N.D.	↑	↑	↑	→	→	↑	→	→	→
aPVAT	HF	N.D.	↑	↑	↑	↑	↑	↑	↑	→	→
	HS	N.D.	→	→	→	→	→	→	→	↓	→
‘↑’ or ‘↓’ indicates *P* < 0.05 versus the control group. N.D.: Not determined.											
(b) Effects of high-fat and high-sucrose diets on the aortic wall											
		Collagen	CD68	MCP-1	MMP-9	MMP-2	TNF-α	Resistin	Angiotensinogen	Adiponectin	MMP-12
Thoracic aorta	HF	→	→	→	→	→	→	→	→	↓	→
	HS	↓	↑	↑	↑	↑	→	→	→	↓	→
Abdominal aorta	HF	↓	↑	↑	↑	↑	↑	→	↑	→	→
	HS	→	→	→	→	→	→	→	↑	→	→
‘↑’ or ‘↓’ indicates *P* < 0.05 versus the control group.											

In this study, we showed the different physiological effects of diets on PVAT and aortic walls. The potential mechanisms underlying these effects may be related to the different adipocyte species that comprise tPVAT and aPVAT. In future studies, we plan to further our understanding of the underlying mechanism of the differential effects of HS and HF diets on adipocytes. Limitation of this study is the lack of data to compare several HF or HS conditions. In this study, we did not estimate the effect of essential fatty acid on PVATs. In addition, complementary quantitative data such as western blotting or real-time PCR are needed to support our histological data. Further studies are needed to clarify the relationship between diets and PVAT.

## Materials and methods

### Animals

Prior to start the experiment, all animal experiments were approved by the Kindai University Animal Care and Use Committee and were carried out according to the Kindai University Animal Experimentation Regulations (Approval number; KAAG-31-006). Male Sprague-Dawley rats aged 6 weeks (SHIMIZU Laboratory Supplies Co., Ltd, Kyoto, Japan) were maintained on a 12 h light/dark cycle with control the temperature about 25 ± 1°C. Twenty rats were divided into three groups, a control group(n = 6), a high-fat group (n = 7), and a high-sucrose group (n = 7), and each group of rats were fed control diet, high-fat diet, and high sucrose diet until the end of the experiment. The diet composition is shown in Table 2. After five weeks of experiment, all the animals were euthanized under anaesthesia.

**Table 2.** Diet composition

(a) Diet composition (g or kcal) of the control, High-Fat (HF), and High-Sucrose (HS) diets.						
Ingredient	control (g)	HF (g)	HS (g)	control (kcal)	HF (kcal)	HS (kcal)
Casein	200	200	200	800	800	800
Maltodextrin	125	125	0	500	500	0
Sucrose	68.8	68.8	700	275	275	2800
Cellulose	50	50	50	0	0	0
AIN-93 G mineral	32	32	32	0	0	0
Dibasic calcium phosphate	16.4	16.4	16.4	0	0	0
AIN-93 vitamin mix	10	10	10	40	40	40
Cystine	3	3	3	12	12	12
Choline chloride	2	2	2	0	0	0
Cornstarch	506.2	0	0	2025	0	0
Lard	20	245	20	180	2205	180
soybean oil	25	25	25	225	225	225
Total	1058.4	777.2	1058.4	4057	4057	4057
(b) Energy composition (kcal%) of the control, High-Fat (HF), and High-Sucrose (HS) diets.						
	control (kcal%)		HF (kcal%)		HS (kcal%)	
Protein	20		20		20	
Carbohydrate	70		20		70	
Fat	10		60		10	
Total (%)	100		100		100	

### Serum glucose, cholesterol and triglyceride level

At the 5 weeks of the experiment, blood (100 μL) was taken from teil vein under anaesthesia (50 mg/kg pentobarbital, i.p.) to determine serum glucose, triglyceride and total cholesterol level. For serum preparation, the blood was centrifuged at 3000 × g for 10 minutes. Then, serum glucose, triglycerides and total cholesterol were measured using commercial kits (Wako Pure Chemical industries, Osaka, Japan) and using the methodology according to the manufacturer instruction. Serum glucose was measured immediately after the sampling. Serums were stored in −80°C until triglycerides and total cholesterol were measured. Serum leptin and insulin were quantified using commercial ELISA Kit (Morinaga Institute of Biological Science, Kanagawa, Japan).

### Sample collection

The thoracic and abdominal aortas were collected with their respective PVAT and fixed with 4% paraformaldehyde. All samples were then dehydrated and embedded in paraffin.

### Histological analysis

The isolated aorta and PVAT was serially sectioned for 10 µm thickness using a microtome (ERM-230 L, Erma, Japan) and stained with haematoxylin and eosin (H&E), picrosirius red (PSR) staining and immunohistochemical stains. ImageJ software (National Institutes of Health, Bethesda, Maryland, USA) was used for quantitative analysis of the stained sections. The area of positive staining in immunohistochemistry was calculated by binarizing the image into black and white using ImageJ. In the semi-quantitative analysis of immunohistochemistry, the area of the adipose tissue section excluding the lipid droplets was defined as the total area, the positive areas were quantified as a percentage.

### Immunohistochemical staining

The deparaffinized tissue sections were permeabilized with 1% Triton X-100 in phosphate-buffered saline (PBS). Then, the sections were blocked for endogenous peroxidase by soaking sections in 3% hydrogen peroxide in methanol for 8 min. The sections were blocked for non-specific binding in blocking solution (Nacalai Tesque, INC, Kyoto, Japan) for 30 min at room temperature. After blockings, the anti-UCP-1 (1:100), anti-CD68 (1:50), anti-MCP-1 (1:100) and MDA (1:100) were applied to the tissue sections for overnight. Then, the sections were washed with PBS and incubated with the horseradish peroxidase (HRP)-conjugated goat anti-rabbit Immunoglobulin G (IgG) diluted at 1:500 or 1000 in blocking solution for 30 min. The DAB kit (Vector Laboratories, Burlingame, CA, USA) was then added on the sections until the desired colour was developed. The stained sections were observed and captured under CX21 LED Olympus microscope (Olympus, Tokyo, Japan) fitted with a digital camera. ImageJ software (National Institutes of Health, Bethesda, Maryland, USA) was quantified the intensity of immunohistochemical stained.

### Statistical analyses

The experimental data was expressed as the mean ± standard error of mean (S.E.M). Statistical differences were evaluated by the Tukey-Kramer test, and a p-value <0.05 was considered statistically significant.

## Supplementary Material

Supplemental Material

## Data Availability

The authors confirm that the data supporting the findings of this study are available within the article and its supplementary materials.
