# Supplementary material for: Different effects of high-fat and high-sucrose diets on the physiology of perivascular adipose tissues of the thoracic and abdominal aorta
Source: Adipocyte. 2021 Sep 13;10(1):412–23. doi: 10.1080/21623945.2021.1965333 (PMC8451459; doi:10.1080/21623945.2021.1965333)
Supplement: Supplemental Material [file KADI_A_1965333_SM6155.zip › suppl/210731 Supplemental Material.docx]

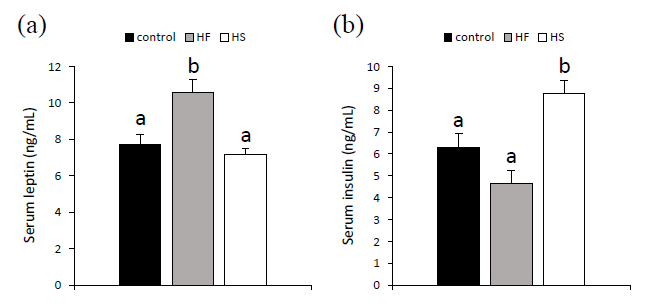


**Supplementary Figure 1**

Effects of high-fat and high-sucrose diets on serum leptin and serum insulin levels. (a) Serum leptin and (b) serum insulin in the three experimental groups. Data are presented as the mean ± standard error of mean (S.E.M). Values with different letters are significantly different (p<0.05).


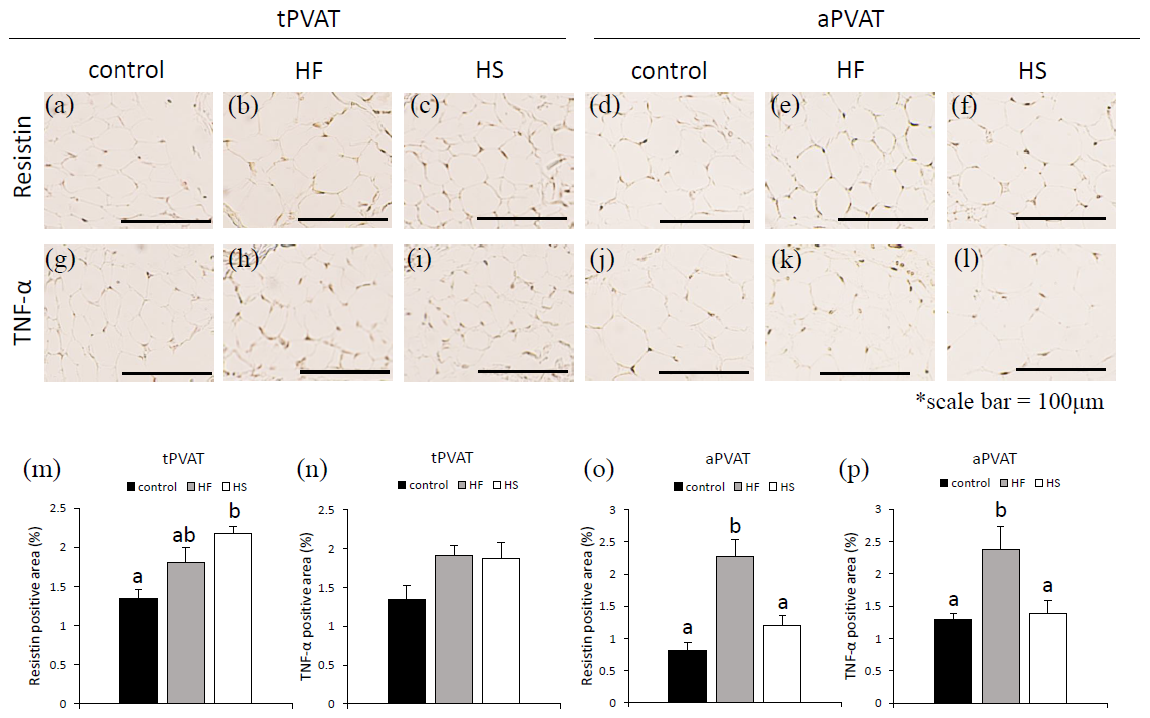


**Supplementary Figure 2**

Effects of high-fat and high-sucrose diets on resistin and TNF-α in PVAT. (a-c) Represented resistin immunostaining of the tPVAT, (d-f) represented resistin immunostaining of the aPVAT, (g-i) represented TNF-α immunostaining of the tPVAT and (j-l) represented TNF-α immunostaining of the aPVAT in the three experimental groups. (m) Quantification of resistin positive areas of the tPVAT, (n) quantification of TNF-α positive areas of the tPVAT, (o) quantification of resistin positive areas of the aPVAT and (p) quantification of TNF-α positive areas of the aPVAT in the three experimental groups. Scale bar = 100μm. Data are presented as the mean ± standard error of mean (S.E.M). Values with different letters are significantly different (p<0.05).


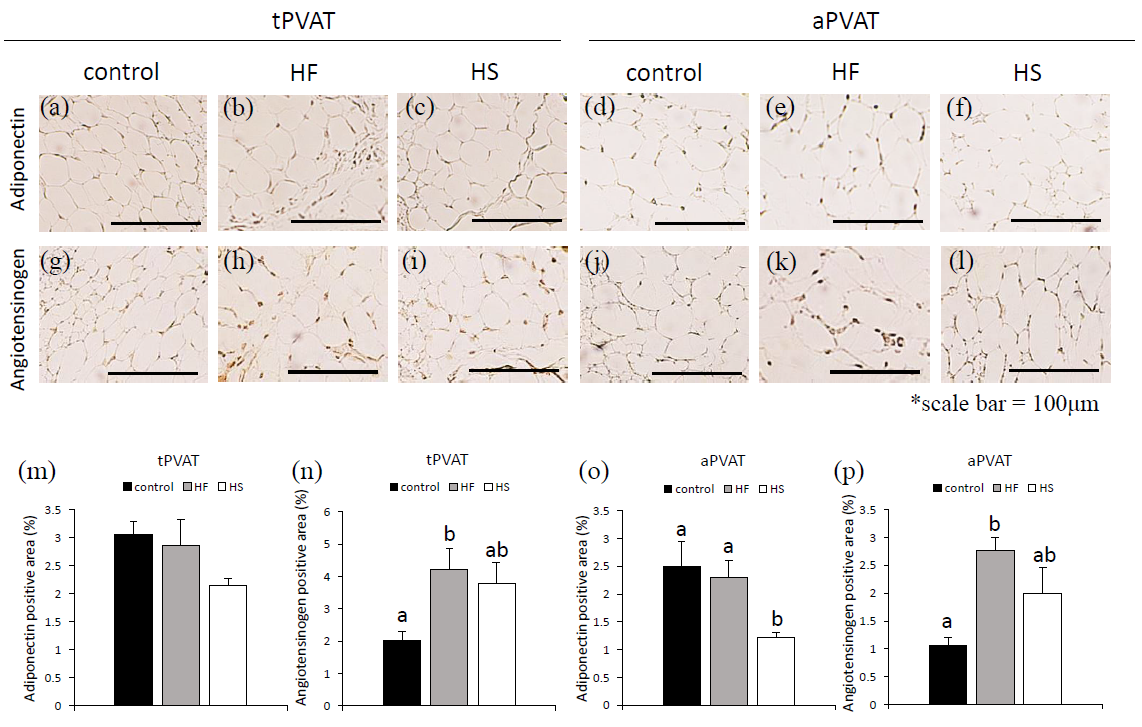


**Supplementary Figure 3**

Effects of high-fat and high-sucrose diets on adiponectin and angiotensinogen in PVAT. (a-c) Represented adiponectin immunostaining of the tPVAT, (d-f) represented adiponectin immunostaining of the aPVAT, (g-i) represented angiotensinogen immunostaining of the tPVAT and (j-l) represented angiotensinogen immunostaining of the aPVAT in the three experimental groups. (m) Quantification of adiponectin positive areas of the tPVAT, (n) quantification of angiotensinogen positive areas of the tPVAT, (o) quantification of adiponectin positive areas of the aPVAT and (p) quantification of angiotensinogen positive areas of the aPVAT in the three experimental groups. Scale bar = 100μm. Data are presented as the mean ± standard error of mean (S.E.M). Values with different letters are significantly different (p<0.05)


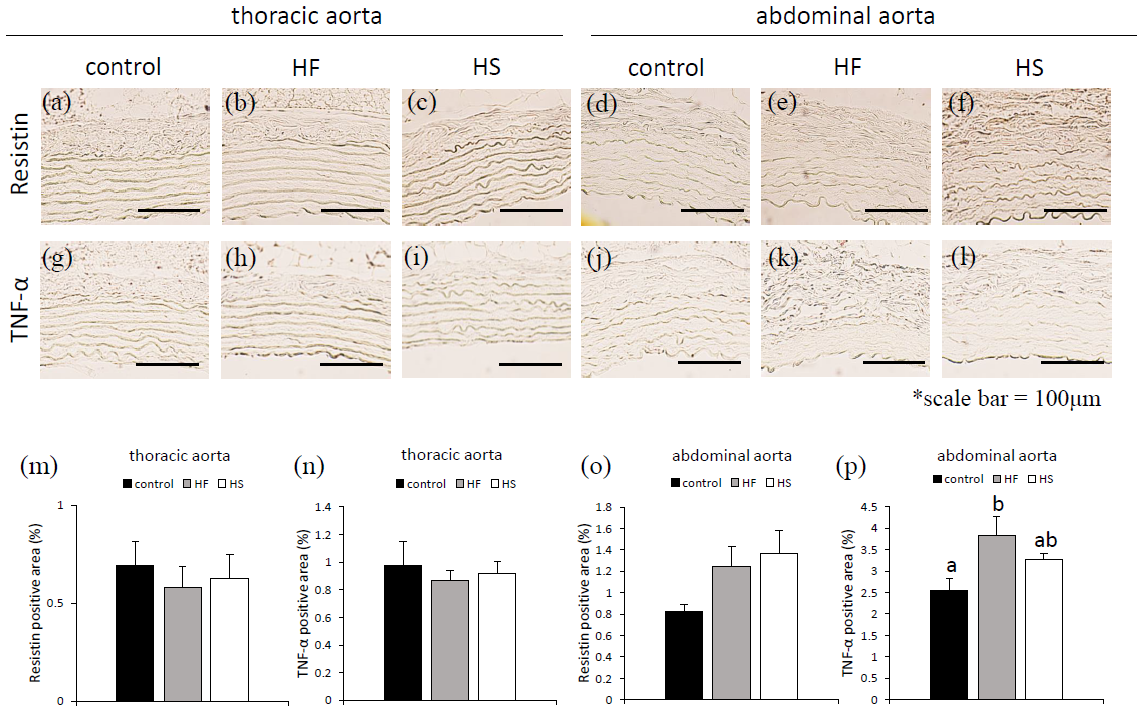


**Supplementary Figure 4**

Effects of high-fat and high-sucrose diets on resistin and TNF-α in the thoracic and abdominal aorta. (a-c) Represented resistin immunostaining of the thoracic aorta, (d-f) represented resistin immunostaining of the abdominal aorta, (g-h) represented TNF-α immunostaining of the thoracic aorta and (j-l) Represented TNF-α immunostaining of the abdominal aorta in the three experimental groups. (m) Quantification of resistin positive areas of the thoracic aorta, (n) quantification of TNF-α positive areas of the thoracic aorta and (o) quantification of resistin positive areas of the abdominal aorta and (p) quantification of TNF-α positive areas of abdominal aorta in the three experimental groups. Scale bar = 100μm. Data are presented as the mean ± standard error of mean (S.E.M). Values with different letters are significantly different (p<0.05).


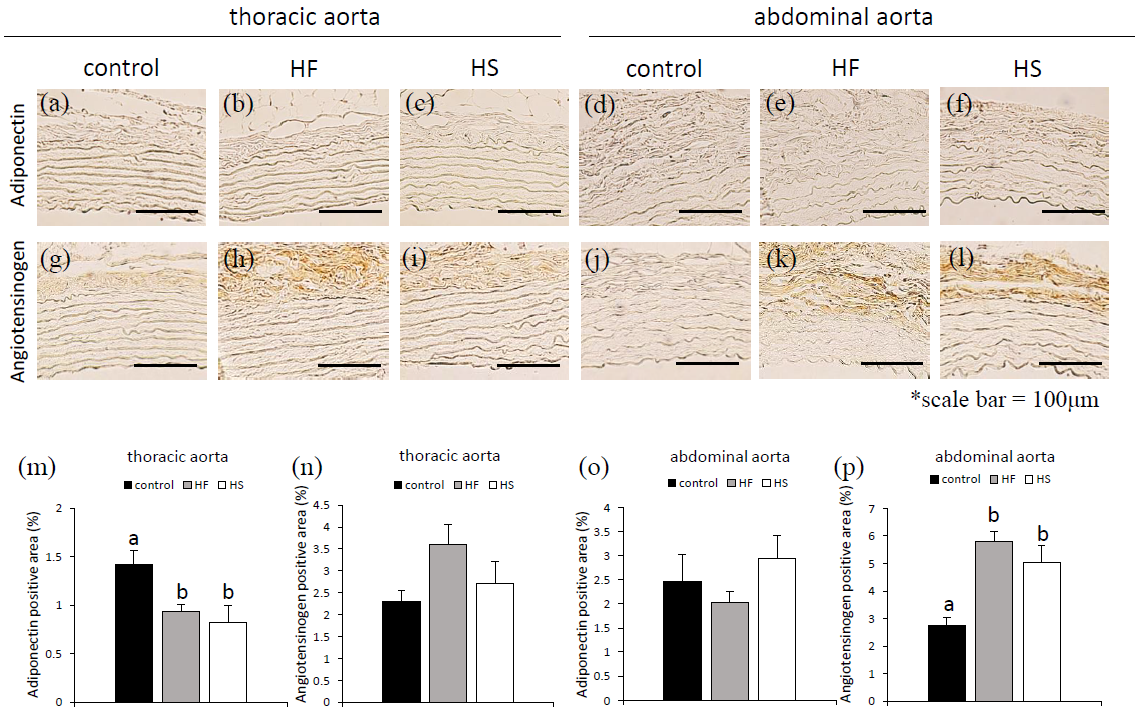


**Supplementary Figure 5**

Effects of high-fat and high-sucrose diets on adiponectin and TNF-α in the thoracic and abdominal aorta. (a-c) represented adiponectin immunostaining of the thoracic aorta, (d-f) represented adiponectin immunostaining of the abdominal aorta, (g-h) represented angiotensinogen immunostaining of the thoracic aorta and (j-l) Represented angiotensinogen immunostaining of the abdominal aorta in the three experimental groups. (m) Quantification of adiponectin positive areas of the thoracic aorta, (n) quantification of angiotensinogen positive areas of the thoracic aorta and (o) quantification of adiponectin positive areas of the abdominal aorta and (p) quantification of angiotensinogen positive areas of abdominal aorta in the three experimental groups. Scale bar = 100μm. Data are presented as the mean ± standard error of mean (S.E.M). Values with different letters are significantly different (p<0.05).


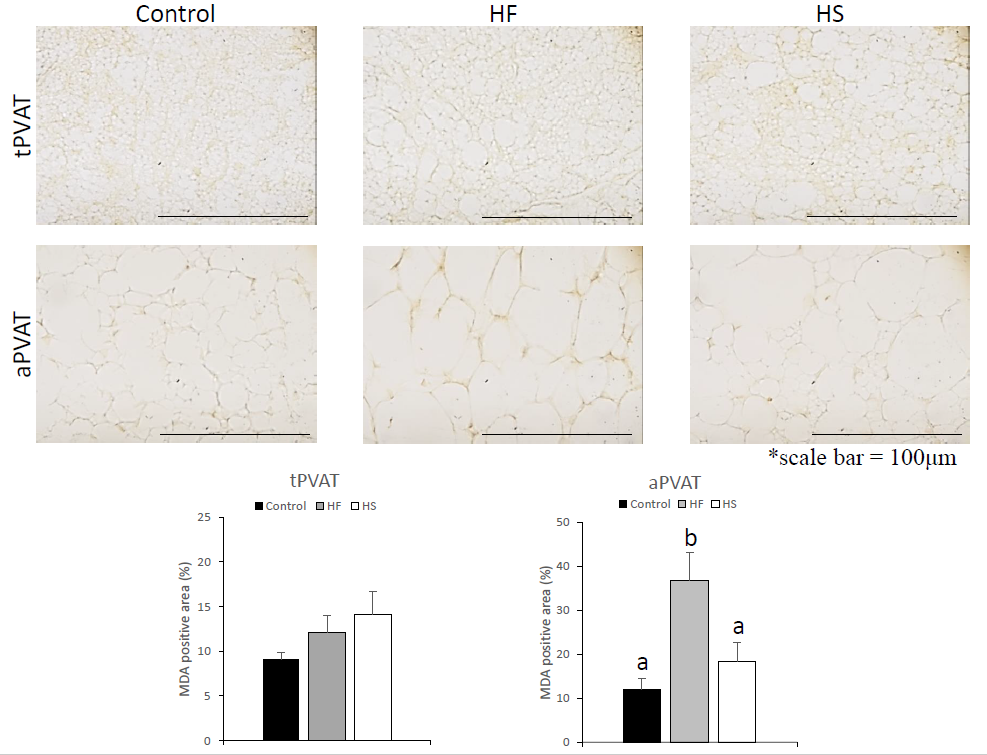


**Supplementary Figure 6**

Effects of high-fat and high-sucrose diets on MDA in PVAT.


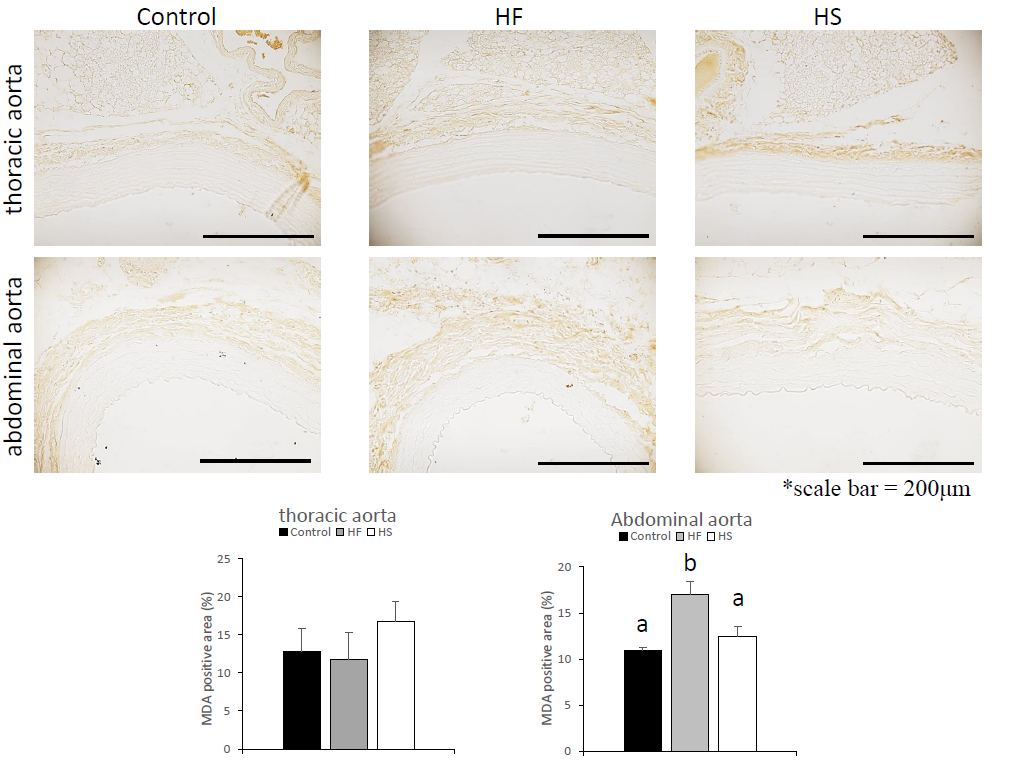


**Supplementary Figure 7**

Effects of high-fat and high-sucrose diets on MDA in aortae.
