# Supplementary figures and images for: Different effects of high-fat and high-sucrose diets on the physiology of perivascular adipose tissues of the thoracic and abdominal aorta
Source: Adipocyte. 2021 Sep 13;10(1):412–23. doi: 10.1080/21623945.2021.1965333 (PMC8451459; doi:10.1080/21623945.2021.1965333)

Supplementary Figure 2

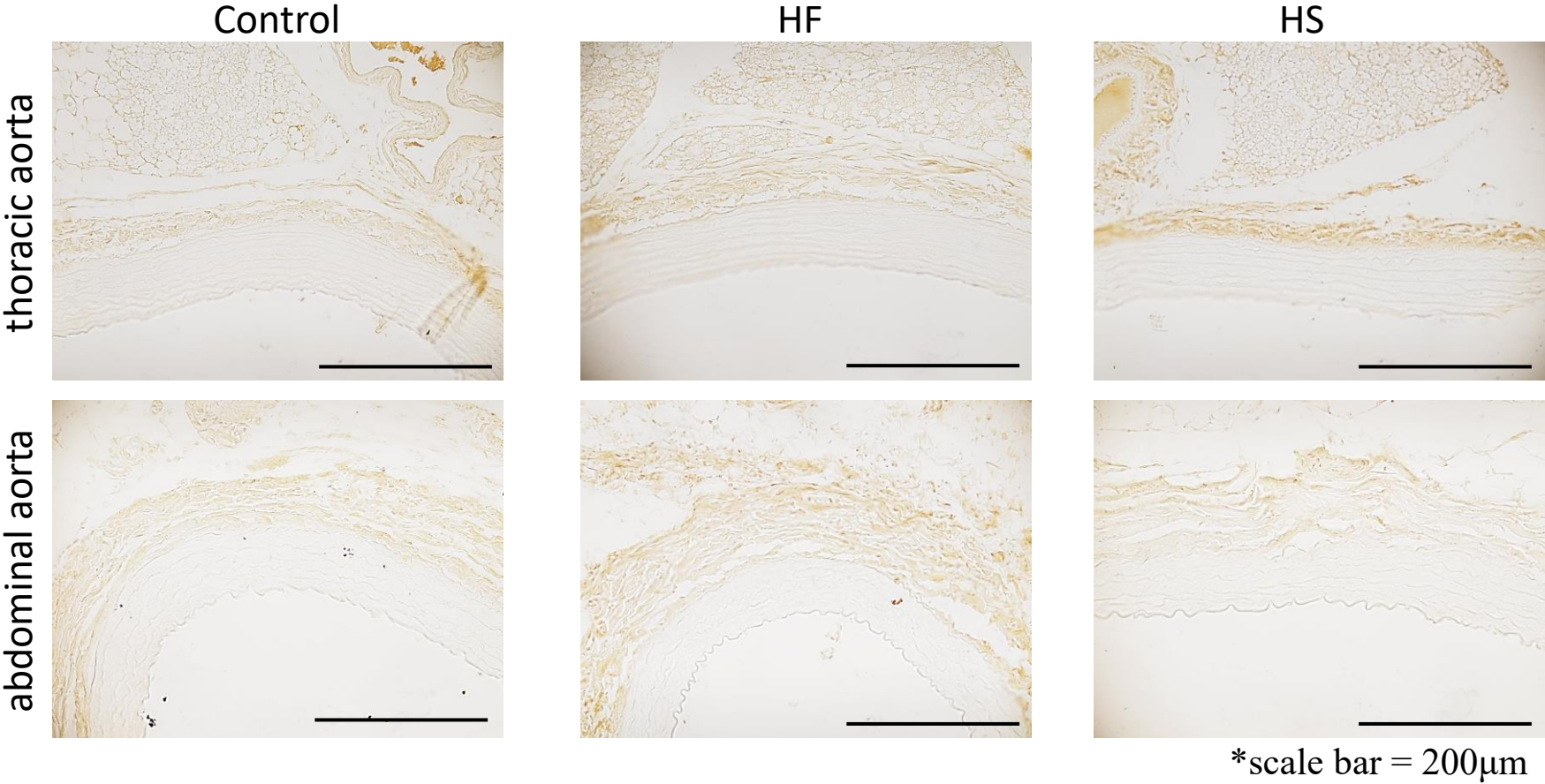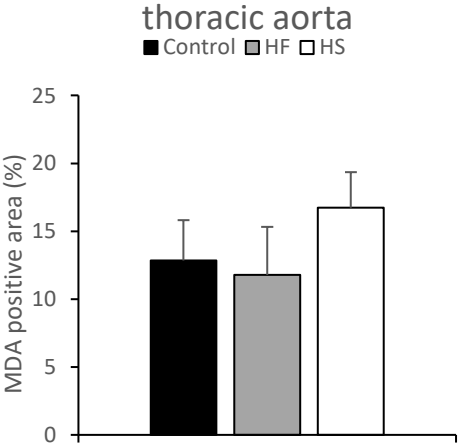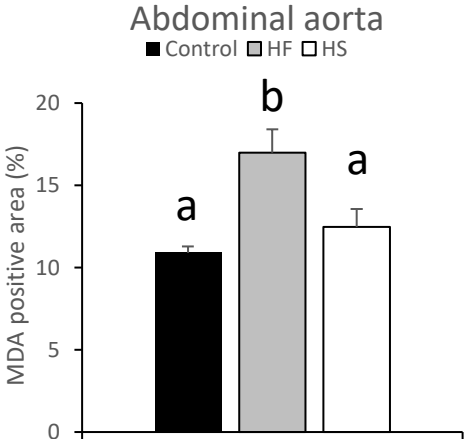

Supplement: Supplemental Material [file KADI_A_1965333_SM6155.zip › suppl/downloadFromZipFile..pdf]

Supplementary Figure 1

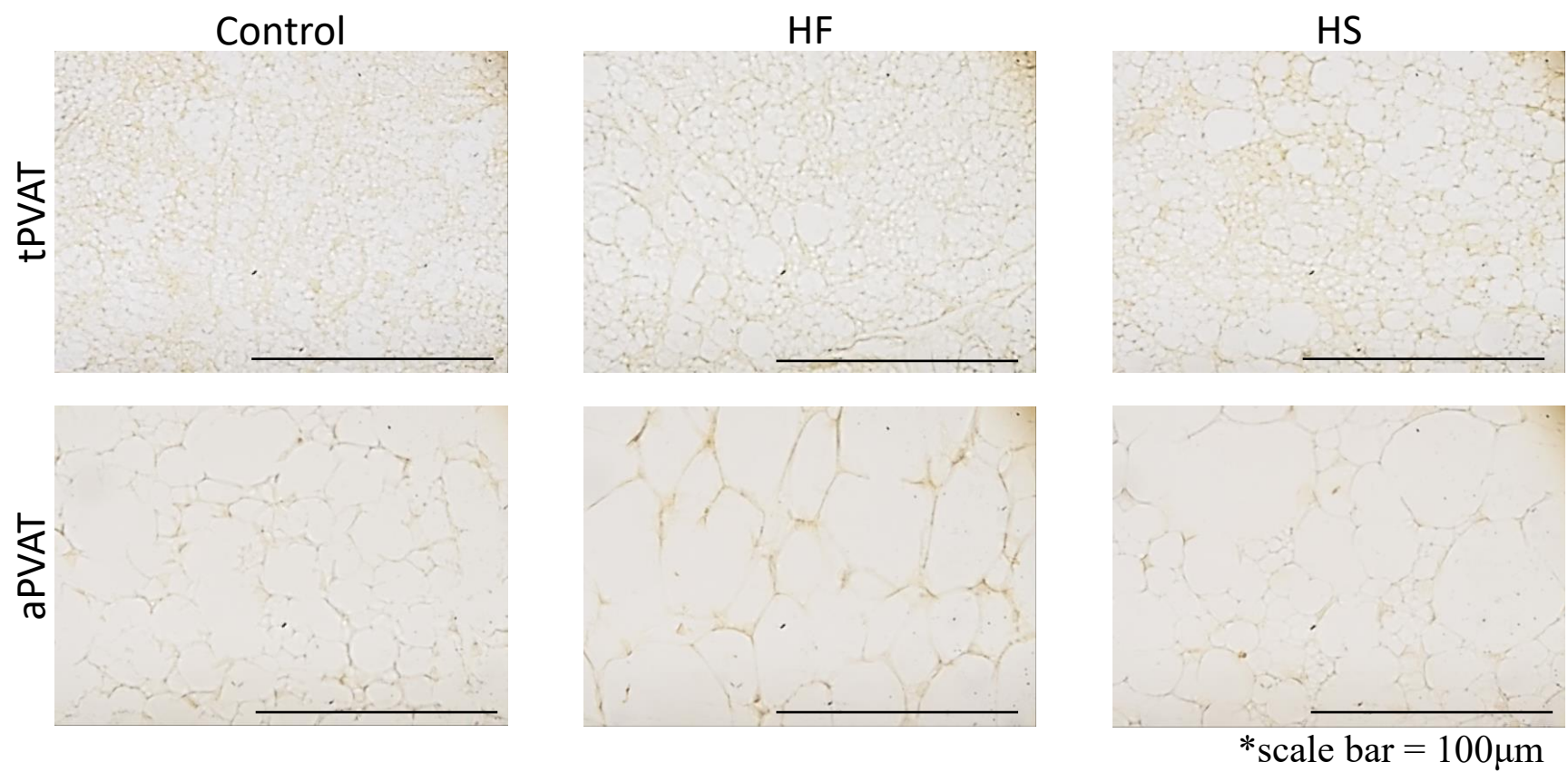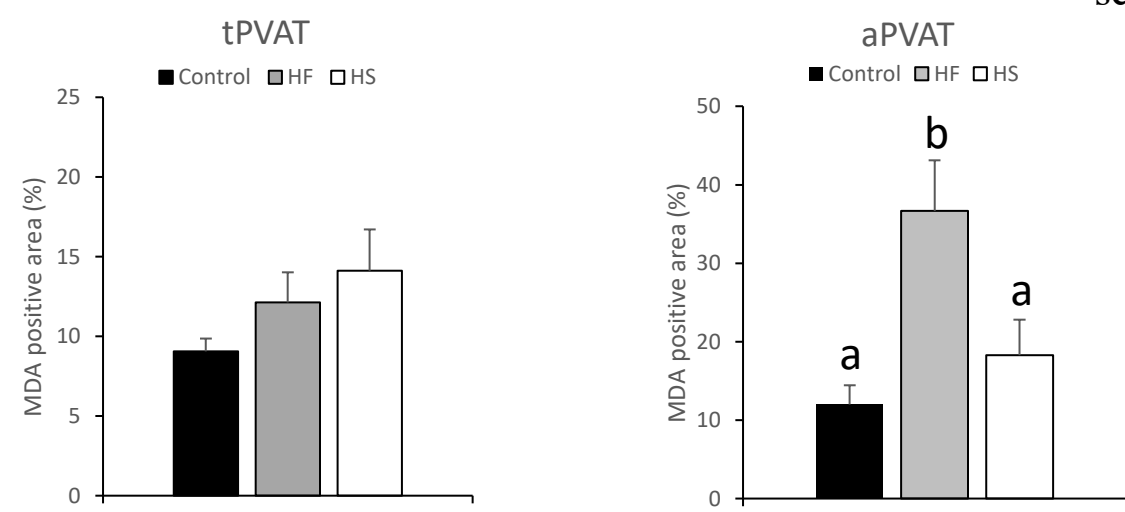

Supplement: Supplemental Material [file KADI_A_1965333_SM6155.zip › suppl/downloadFromZipFile.pdf]
